# Dynamic Plasticity Model for Rapidly Heated 1045 Steel Up to 1000 °C

**DOI:** 10.6028/jres.126.026

**Published:** 2021-10-27

**Authors:** Steven P. Mates, Sheng-Yen Li

**Affiliations:** 1 National Institute of Standards and Technology, Gaithersburg, MD 20899, USA; 2 Southwest Research Institute, San Antonio, TX 78238, USA

**Keywords:** carbon steel, constitutive model, high strain rate, high temperature, machining simulations

## Abstract

The National Institute of Standards and Technology (NIST) developed an experimental technique to measure the dynamic flow stress of metals under rapid heating to study their time-dependent plastic response when heating times are short enough to interrupt or bypass thermally driven microstructural evolution. Such conditions may exist as chips are formed in the machining process. Measurements of American Iron and Steel Institute1045 steel behavior up to 1000 °C showed complex thermal softening due to dynamic strain aging effects and the diffusion-limited austenite transformation process beginning at the A1 temperature (712 °C). This paper proposes a constitutive model to capture the flow stress and hardening evolution of 1045 steel under rapidly heated conditions for simulating metal cutting. The model combines the Preston-TonksWallace plasticity model, which uses five parameters to capture complex rate- and temperature-sensitive strain hardening, with a dual-ratesensitivity model to capture the response of rapidly heated 1045 steel. Finally, a strain-rate-dependent Gaussian function is introduced to capture dynamic strain aging effects, which act over a narrow range of temperatures that change with strain rate. The proposed model is compared to existing plasticity models for 1045 steel over the range of data available and at a representative machining condition.

The National Institute of Standards and Technology (NIST) developed an experimental technique to measure the dynamic flow stress of metals under rapid heating to study their time-dependent plastic response when heating times are short enough to interrupt or bypass thermally driven microstructural evolution. Such conditions may exist as chips are formed in the machining process. Measurements of American Iron and Steel Institute1045 steel behavior up to 1000 °C showed complex thermal softening due to dynamic strain aging effects and the diffusion-limited austenite transformation process beginning at the A1 temperature (712 °C). This paper proposes a constitutive model to capture the flow stress and hardening evolution of 1045 steel under rapidly heated conditions for simulating metal cutting. The model combines the Preston-Tonks-Wallace plasticity model, which uses five parameters to capture complex rate- and temperature-sensitive strain hardening, with a dual-rate-sensitivity model to capture the response of rapidly heated 1045 steel. Finally, a strain-rate-dependent Gaussian function is introduced to capture dynamic strain aging effects, which act over a narrow range of temperatures that change with strain rate. The proposed model is compared to existing plasticity models for 1045 steel over the range of data available and at a representative machining condition.

## 1 Introduction

The National Institute of Standards and Technology (NIST) developed an experimental technique to measure the dynamic compressive flow stress of metals undergoing rapid pre-heating to explore the time-dependent plastic response of metals caused by heating times that are too short for microstructures to reach equilibrium or quasi-equilibrium. Nonequilibrium behavior may occur when a metal is rapidly heated to temperatures where its microstructure becomes thermodynamically unstable and begins to transform with kinetics that are slow relative to the heating time. In machining American Iron and Steel Institute (AISI) 1045 steel, hereafter called 1045 steel, for example, workpiece heating that occurs by adiabatic plastic deformation and friction can generate temperatures well above those needed to induce thermally driven microstructural evolution. Two effects are possible: subcritical annealing, starting near 540 °C [1] in cold-worked material, and austenite transformation at the A1 temperature, which is just above 700 °C, depending on the composition of the steel. In high-speed machining of 1045 steel (300 m/min cutting velocity), while temperatures are limited to about 400 °C in the primary shear zone, they can greatly exceed A1 in the secondary shear zone along the rake face, as measured by thermocouples [2] and infrared thermography [3]. As such, plasticity models developed from data obtained under furnace preheating, where heating times are much longer than those in machining, will ignore possible time-dependent effects that may be present during real machining processes.

**Fig. 1:**
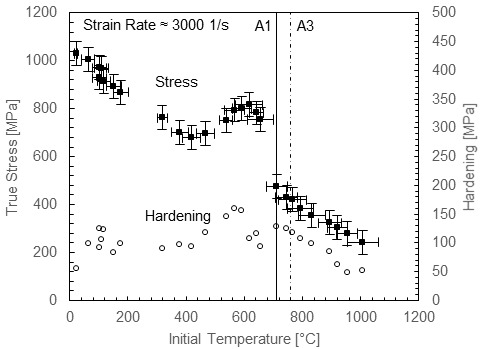

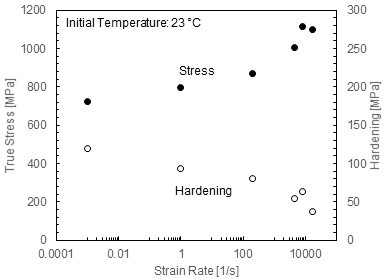
Compression behavior of 1045 ferrite-pearlite steel at high strain rate up to 1000 °C (left) and at strain rates to 20 000 1/s at room temperature (right) at 0.1 plastic strain (hardening evaluated between 0.075 and 0.2 strain). Error bars are from Ref. [4]. Dynamic experiments without error bars have similar uncertainties to dynamic experiments with error bars. Quasi-static experimental uncertainties are considerably less.

Figure 1 shows the dynamic flow stress behavior of annealed ferrite-pearlite 1045 steel under rapid heating [4], where dynamic strain aging effects and austenite transformation produce large excursions in plastic flow stress as well as hardening behavior with temperature. Strain hardening has an important role in chip formation mechanics, and it is therefore important to capture these effects in a constitutive model [5]. Complex hardening behavior cannot be captured with general-purpose plasticity models, such as the Johnson-Cook (JC) model [6], the Zerilli-Armstrong (ZA) model [7], or the power law (PL) model [8], which show broad agreement with many metals but have too few parameters to handle the complex hardening of 1045 steel over wide ranges of conditions. For example, the rate sensitivity of carbon steel does not fall in line with the simple logarithmic behavior assumed in the JC model for the range of strain rates relevant to machining. Its strain rate sensitivity has been observed to increase significantly at high strain rates in low-carbon annealed steel [9], low-carbon cold-rolled steel [10] and annealed 1045 steel [11], and strain hardening has been observed to decrease at higher strain rates due to adiabatic thermal softening in compression [10] and torsion, as shown by several studies, including Ref. [12]. Basic plasticity models can be, and have been, modified to capture some of these features, which are thought to be important to carbon steel machining behavior, including rate-modified hardening and dynamic strain aging [13].

Turning back to non-equilibrium behavior of steel under rapid heating, an important early investigation was performed using fast induction heating (5 s heating time) combined with Kolsky bar compression testing [14]. These experiments revealed behavior similar to that shown in Fig. 1, and the results were used to develop a modified PL plasticity model for machining simulations [2]. However, this earlier data set was limited to a maximum temperature of 800 °C, with 100 °C increments, whereas the newer data set, having much finer temperature resolution, particularly near the A1 temperature, presents a more detailed picture of the behavior of thermal softening in carbon steel. For example, it reveals a more sudden collapse of the flow stress at the A1 temperature where pearlite decomposes to austenite compared with the seemingly more gradual reduction suggested by the less-well-resolved data set. The newer picture of 1045 steel behavior near A1 yields a better sense of the importance of austenite transformation kinetics and the dynamic strain aging (DSA) effect and their influence on plasticity in this critical region. Austenite transformation kinetics will affect the slope of the drop in flow stress with temperature; *e.g.*, shorter heating times presumably mean less transformation and a more gradual stress reduction. DSA amplifies the flow stress just below the A1 temperature, leading to a massive drop as the pearlite dissolves, as shown in Fig. 1. DSA strengthening comes from the diffusion of interstitial carbon and nitrogen to mobile dislocation cores, creating a Cottrell atmosphere [15] that effectively pins dislocations, requiring higher stresses to break them free again. The peak DSA temperature depends on strain rate, and in Kolsky bar tests, it occurs near 600 °C, *i.e.*, only about 100 °C below A1. Under static loading, strain aging is known to cause upper-lower yield point behavior in steel, along with the Portevin–Le Chatelier (PLC) band instability [16]. However, under dynamic loading, these effects are suppressed, because there is not time for the interstitial solutes to accumulate around fast-moving mobile dislocations [17], and when the immobile dislocations remain locked, the overall dislocation density is forced to increase more rapidly, enhancing strain hardening [18]. Strain rate influences both the amplitude of the DSA hardening increase and the peak DSA temperature in carbon steel in both tension [19] and compression [20], although in torsion experiments on hot-rolled low-carbon steel, the effective magnitude decreases rather than increases with increasing strain rate [21]. DSA also occurs over a finite range of temperatures, with “cut-on” and “cut-off” temperatures that are gradual rather than sharp. As discussed, experiments show that these effects rapidly disappear above A1 under dynamic loading (Fig. 1), further modifying plastic flow and hardening at this temperature. Another interesting feature of DSA is that, when it is active, the kinetics are very rapid, as has been explored in extremely rapid (less than 0.1 s) heating tests [14]. This more detailed picture of the plastic flow behavior of carbon steel over wide ranges of temperature and strain rate requires a nimble constitutive model framework that can be customized to capture hardening evolution with strain rate and temperature as well as dynamic strain aging and phase transformation effects.

**Fig. 2:**
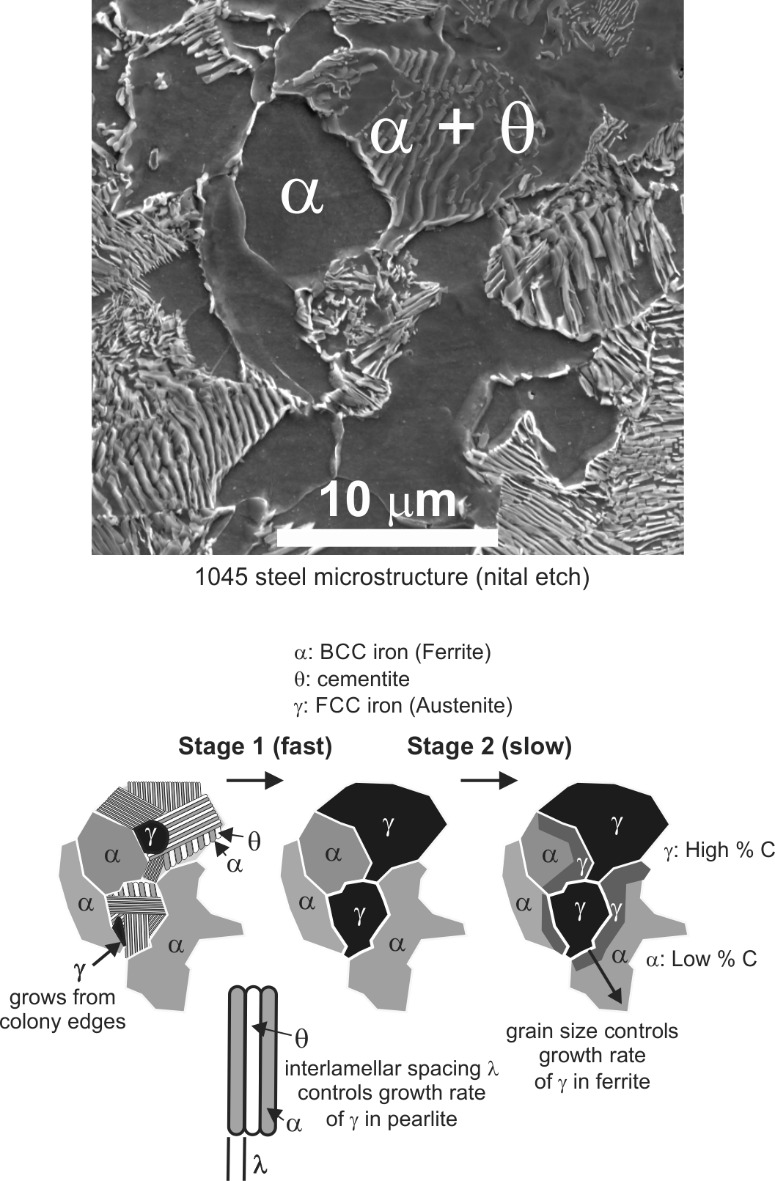
Diffusion-controlled austenite transformation process in ferrite-pearlite carbon steel.

A model that can capture the austenite transformation effect on the flow stress must consider how the process occurs in two stages with different kinetics for each stage. The two stages are shown schematically in Fig. 2. Austenite begins to form at pearlite colony boundaries and quickly consumes them in a matter of seconds, with kinetics that depend on the carbon diffusion rate under the applied thermal history, on the fineness of the lamellae spacing [22], and on alloy content like Mn and Si [23]. Austenite, having a face-center-cubic (FCC) lattice, exhibits very different plastic behavior compared to pearlite colonies, which consist of alternating plates of body-center-cubic (BCC) iron and orthorhombic cementite (Fe_3_C). The yield and hardening behavior of FCC and BCC metals is usually quite different [24]. The second stage involves the much slower growth of austenite into the remaining ferrite, the kinetics of which depend on carbon diffusion into the BCC lattice according to microstructural studies [25] and *in situ* X-ray diffraction measurements [26]. In the intercritical region between the A1 and A3 temperatures (A3 = 760 °C for the 1045 steel investigated here; A1 and A3 can vary by small amounts depending on the alloy composition), austenite and ferrite exist together under equilibrium conditions, while only austenite exists at equilibrium above A3. Upon heating to A1 over the course of several seconds, pearlite transforms to austenite under para-equilibrium conditions, but full equilibrium is not yet achieved. Due to limited diffusion time, the newly formed austenite fraction is smaller and contains more carbon compared to equilibrium, and the ferrite fraction is correspondingly larger. This para-equilibrium microstructure is believed to cause the difference in dynamic flow stress behavior above A1 compared to equilibrium models [4]. We note finally that at very high heating rates (>100 °C/s), new microstructural evidence suggests that austenite can form through a faster, diffusionless transformation mechanism [27], where phase fractions and carbon distributions may deviate from equilibrium in yet a different manner than so far described. High heating rates also shift the transformation temperatures to higher values compared to equilibrium.

In machining, the importance of a thermally evolving microstructure, to include annealing or austenite formation or both, depends critically on time as well as temperature: Is the workpiece/chip at temperature for enough time to induce transformation, and if it is, how far does that transformation proceed? To study this in the laboratory, measurements of the plastic flow stress are needed at heating times that span the transformation times. For carbon steels, subcritical annealing (below A1) can occur in seconds to minutes or longer as revealed by hardness measurements [14] and a nondestructive magnetic (coercive field) technique [28]. The initial stage of austenite formation is much faster and difficult to interrupt even with rapid heating methods, and analysis of the transformation kinetics is complicated by the fact that thermal gradients are present in the test samples [29]. Despite its speed, the first stage may yet require more time than is available during high-speed machining (<1 ms). It may therefore be unlikely for pearlite to have enough time to decompose even partially, unless the faster diffusionless transformation mechanism is involved. Evidence of austenite transformation in machined chips is rare, although a diffusionless transformation may leave little microstructural evidence behind [27]. That said, even if pearlite does not have time to transform, a more gradual drop in the flow stress around A1 might be expected due to the “cut-off” of dynamic strain aging effects, if the combination of strain rate and temperature during chip formation is in the correct range. The kinetics of DSA appear to be quite rapid, but they are not well understood. It is possible that data obtained with heating times of seconds above the A1 temperature would fall in line with workpiece behavior in machining, with pearlite in the machined workpiece being replaced by austenite in laboratory experiments with similar strength levels to pearlite (*e.g.*, pearlite being no longer strengthened by DSA). This remains a matter of speculation. Regardless, the complex two-stage austenite transformation process, combined with DSA behavior near this temperature, is quite beyond the capacity of general-purpose plasticity models to capture without significant modification, such as in Ref. [13], with the result that these material effects are often ignored in machining simulations.

In this paper, a modeling framework is described to capture the dynamic behavior of 1045 carbon steel up to 1000 °C under rapid heating and over a wide range of strain rates at room temperature, including the effects of dynamic strain aging on yield and hardening behavior and the effects of phase transformation above the A1 temperature. Data showing the effect of temperature on strain rate are not available at present, but at high strain rates, temperature has little effect on rate sensitivity for BCC iron [30]. Above A1, where FCC iron exists, the rate sensitivity may increase, but data are needed at short heating times to explore this issue. The model framework is based on the viscoplastic constitutive model developed by Preston, Tonks, and Wallace (PTW model) [31], which offers much more flexibility to model hardening evolution with temperature and strain rate compared to general-purpose plasticity models. In our approach, we did not attempt to model individual phases explicitly, as do some microstructure-level machining models [32], although in the future, this approach may be considered, especially when enough data are available to accurately track the evolution of phase compositions with time and temperature. We adjusted the basic PTW model to capture carbon steel behavior in two ways: First, the rate sensitivity was modified to capture the strong upturn in sensitivity in a way that also allowed for temperature sensitivity to be captured in the plasticity transition region of strain rates in the original PTW model. This was accomplished by using the dual-rate-sensitivity model developed by Vural *et al*. for low-carbon steel [10]. Second, a Gaussian term was added to capture DSA effects, including the effects of temperature and strain rate on the peak temperature and stress and hardening magnitudes. A similar additive Gaussian function was used in a model of the blue brittle effect in martensitic steel [33]. Our model was calibrated using the data set partially shown in Fig. 1 (full stress-strain curves were used for model calibration). While the present model was developed using data obtained with a single heating time, here 3.5 s, future work will include data for a range of heating times to develop a time-dependent strength model for carbon steel above the A1 temperature. For now, the model will require a suitable transition function to bridge the transformation gap smoothly over a finite range of temperatures. The resulting model fit was compared to two models in the literature used for 1045 steel machining simulations: an unmodified JC model calibrated up to 600 °C [34] and a PL model modified to include dynamic strain aging effects with temperature dependence up to 1200 °C [13]. We finally note that fracture was not considered in this model, although it shows some important effects in steel machining simulation results [35]. The remainder of the paper is organized as follows. First, the modified PTW model is fully described, then the fitting procedure is outlined, and finally the fit is compared to the experimental data and discussed. A list of symbols used is given in Table 1.

## 2 Modified PTW Model for Carbon Steel

**Table 1:** List of symbols and their associated units.

Symbols	Unit	Description
*β*		rate sensitivity exponent
*ε*	m/m	normal strain
$\delta \varepsilon_{i}$	m/m	normal strain increment
*γ*	m/m	shear strain
$\dot{\gamma }$	1/s	shear strain rate
*Δγ*	m/m	shear strain increment
*η*		Taylor-Quinney coefficient
*θ*		Voce hardening modulus
$\dot{\xi }$	1/s	reference shear strain rate
*τ, * $\hat{\tau }$	GN/m^2^, —	shear stress; normalized shear stress
*ρ*	kg/m^3^	density
*σ*	GN/m^2^; —	normal stress; DSA temperature range parameter
*A1, A3*	°C	steel transformation temperatures
*C*		rate sensitivity parameter
*c_p_*	J/kg-K	heat capacity
*G*	GN/m^2^	elastic shear modulus
*k*		thermal sensitivity parameter
*s*		rate sensitivity transition parameter
*T,* $\;\hat{T}$	°C, K/K	temperature; normalized temperature
*y*		rate sensitivity exponent parameter
Subscripts		Description
*0*		zero temperature yield and saturation stress; reference strain rate; initial temperature
*01, 02*		low and high ranges of the reference strain rate, respectively
*1, 2*		low and high ranges of rate sensitivity parameters and rate sensitivity exponent parameters, respectively
*∞*		infinite temperature for yield and saturation stresses
*μ*		mean temperature of dynamic strain aging effects
*DSA; 0,DSA*		dynamic strain aging (DSA); DSA amplitude factor
*i*		normal strain increment index
*m*		melting point
*sat*		saturation stress on the rate sensitivity parameter
*t*		transition shear strain rate in rate sensitivity model
*y, s*		yield stress, saturation stress

The PTW model was originally developed to capture the thermo-viscoplastic behavior of metals over a very wide range of strain rates, up to 10^12^ 1/s, to capture extreme events such as explosive loading and high-velocity impact. The model is based on strain rate– and temperature-sensitive yield (subscript *y*) and saturation (subscript *s*) shear stresses *τ*, where the ^ overbar denotes normalization by the temperature-dependent shear modulus *G*:

**Table:** 

	${\hat{\tau }}_{y}=\frac{\tau_{y}}{G\left(T\right)}=y_{0}-\left(y_{0}-y_{\infty }\right)erf\left[k\hat{T\;}ln\left(\frac{C\dot{\xi }}{\dot{\gamma }}\right)\right]$	(1)
	${\hat{\tau }}_{s}=\frac{\tau_{s}}{G\left(T\right)}=s_{0}-\left(s_{0}-s_{\infty }\right)erf\left[k\hat{T\;}ln\left(\frac{C\dot{\xi }}{\dot{\gamma }}\right)\right]$	(2)
	$\frac{d\hat{\tau }}{d\gamma }=\theta \frac{{\hat{\tau }}_{s}-\hat{\tau }}{{\hat{\tau }}_{s}-{\hat{\tau }}_{y}}$	(3)

Additional subscripts in the equations denote zero (*0*) and infinite (*∞*) temperature, where the latter is not literally infinite but instead refers to the end of thermally activated slip. Also, *erf* refers to the Gaussian error function. Other parameters include the reference and actual shear strain rates $\dot{\xi }$ and $\dot{\gamma }$, while *k*, *C*, and *θ* are fitting constants having to do with thermal, strain rate, and hardening rate effects, respectively. Temperature is normalized by the melting temperature of the material $\hat{T}=\left(T+273\right)/T_{m}=\left(T+273\right)/1705\;K$. Note that $\hat{T}$ has units of K/K and is the only use of Kelvin units in the model, i.e., *T* otherwise has units of °C.

A particular advantage of this model over simpler ones is that the strain hardening and thermal softening behavior, both important to machining simulations, are controlled by five parameters (*y_0_, y_∞_, s_0_, s_∞_, θ*) rather than just two in the basic JC model and the ZA model for BCC materials, and typically two or three parameters for the PL model. The added flexibility requires more extensive calibration data, however. We note that here we used standard Voce hardening rather than the modified hardening model described in Ref. [31] to improve fitting results for FCC metals. Here, the standard Voce hardening model (*p* = 0 in the modified hardening model of the original work) was adequate to fit 1045 data even in the austenite (FCC) regime.

The reference shear strain rate, $\dot{\xi }$, is defined in terms of the inverse of the time required for a shear wave to cross one atom. However, a modification to the model rate sensitivity required to capture steel behavior led us to omit a reference shear strain rate for the following reasons. Rate sensitivity in the original PTW model for strain rates spanning 10^3^ 1/s to 10^12^ 1/s is handled by patching together three different plasticity regimes: thermally activated slip (low to moderate strain rates), overdriven shock (very high strain rates, 10^9^ to 10^12^), and a transition region where the rate sensitivity gradually changes from low to high. In the overdriven shock regime, work hardening and temperature effects are neglected. Thus, the transition behavior in the model goes from high temperature sensitivity but low rate sensitivity to high rate sensitivity and no temperature sensitivity or work hardening. The overdriven shock model reduces to the following expression, where *const* refers to a scaling constant:

**Table:** 

	${\hat{\tau }}_{s}={\hat{\tau }}_{y}=const{\left(\frac{\dot{\gamma }}{\dot{\xi }}\right)}^{\beta }$	(4)

Between the thermal activation and overdriven shock regimes, the PTW model uses a MIN-MAX criterion:

**Table:** 

	${\hat{\tau }}_{y}=MAX\left\{y_{0}-\left(y_{0}-y_{\infty }\right)erf\left[k\hat{T\;}ln\left(\frac{C\dot{\xi }}{\dot{\gamma }}\right)\right],\;MIN\left[y_{1}{\left(\frac{\dot{\gamma }}{C\dot{\xi }}\right)}^{y_{2}},s_{0}{\left(\frac{\dot{\gamma }}{C\dot{\xi }}\right)}^{\beta }\right]\right\}$	(5)
	${\hat{\tau }}_{s}=MAX\left\{s_{0}-\left(s_{0}-s_{\infty }\right)erf\left[k\hat{T\;}ln\left(\frac{C\dot{\xi }}{\dot{\gamma }}\right)\right],s_{0}{\left(\frac{\dot{\gamma }}{C\dot{\xi }}\right)}^{\beta }\;\right\}$	(6)

Two additional parameters are introduced in these equations to capture the upturn in yield rate sensitivity: *y_1_* and *y_2_*. Unfortunately, this approach eliminates the temperature sensitivity of the yield stress in the transition region, *e.g.*, the MIN criterion in Eq. (5), which is inappropriate for carbon steels because the transition region begins at strain rates as low as 100 1/s, where temperature and hardening effects remain important. As a result, the rate sensitivity was here altered by moving the strain rate term outside of the error function and applying it as a multiplicative factor, as is done in the JC model:

**Table:** 

	${\hat{\tau }}_{y}=\left\{y_{0}-\left(y_{0}-y_{\infty }\right)erf\left[k\hat{T}\right]\right\}∙\left(1+C\ln\frac{\dot{\gamma }}{\dot{\gamma_{0}}}\right)$	(7)
	${\hat{\tau }}_{s}=\left\{s_{0}-\left(s_{0}-s_{\infty }\right)erf\left[k\hat{T}\right]\right\}∙\left(1+\frac{C}{C_{sat}}\ln\frac{\dot{\gamma }}{\dot{\gamma_{0}}}\right)$	(8)

*C_sat_* is called the saturation rate sensitivity parameter, and it is added to allow the total hardening capacity (${\hat{\tau }}_{s}-{\hat{\tau }}_{y}$) to be reduced with increasing strain rate (*C_sat_* ≥ 1), which is usually observed in metals, with only one additional parameter needed.

This modification can fit carbon steel behavior by extending the thermally activated region to high enough strain rates to capture machining behavior (≤ 10^6^ 1/s). In addition, we adopted the dual-rate-sensitivity model developed by Vural *et al*. [10], which uses four coefficients to provide a smooth change in rate sensitivity from the low- to high-strain-rate regimes, as follows:

**Table:** 

	$C=C_{1}+\frac{C_{2}-C_{1}}{2}∙\left[1+\tanh\left(s\ln\left[\frac{\dot{\gamma }}{{\dot{\gamma }}_{t}}\right]\right)\right]$	(9)
	${\dot{\gamma }}_{0}={\dot{\gamma }}_{01}+\frac{{\dot{\gamma }}_{02}-{\dot{\gamma }}_{01}}{2}∙\left[1+\tanh\left(s\ln\left[\frac{\dot{\gamma }}{{\dot{\gamma }}_{t}}\right]\right)\right]$	(10)
	${\dot{\gamma }}_{02}=$ ${\left({\dot{\gamma }}_{01}\right)}^{\frac{y_{1}}{y_{2}}}∙{\left({\dot{\gamma }}_{t}\right)}^{\frac{{y_{2}-y}_{1}}{y_{2}}}$*; *$C_{1}=y_{1}{\left(\frac{\dot{\gamma }}{\dot{\gamma_{01}}}\right)}^{y_{1}};C_{2}=y_{2}{\left(\frac{\dot{\gamma }}{\dot{\gamma_{02}}}\right)}^{y_{2}}$	(11)

We fixed the following parameters to values determined in Ref. [10] for a cold-rolled low-carbon steel to reduce the number of fitting parameters: ${\dot{\gamma }}_{01}=5\times 10^{-6}$; *s* = 400; and ${\dot{\gamma }}_{t}=166$. The rate sensitivity was then determined by just two fitting parameters: *y_1_* and *y_2_*. For low-carbon steel, *y_2_* ≈ 3*y_1_* to 5*y_1_*.

We assumed for the sake of model fitting that adiabatic heating occurs when the strain rate exceeds 10 1/s. The adiabatic heating is calculated by numerical integration of the stress-strain curve:

**Table:** 

	$T_{i}=T_{0}+\sum_{0}^{i}\left[\frac{\eta ∙\sigma_{i}∙\delta \varepsilon_{i}}{\rho \left(T_{i}\right)∙c_{p}\left(T_{i}\right)}\right]$	(12)

In the above equation, *i* is the strain increment index, 0 is the initial condition, and *η* is the fraction of plastic work converted into heat, called the Taylor-Quinney coefficient, which is taken to be 0.9.

The temperature dependences of the shear modulus (*G*), heat capacity (*c_p_*), and density (*ρ*) of 1045 steel were calculated from equilibrium thermodynamics using commercial software (*T* in °C):

**Table:** 

$G=82.218-0.012085T-5.7217\times 10^{-5}T^{2}+2.6635\times 10^{-8}T^{3}$ * [GPa] for all T*
$c_{p}=506.0+0.3512T+4.951x10^{-4}T^{2}\;$[J/kgK] for C ≤ A1
$c_{p}=470.18+0.1142T\;$[J/kgK] for T > A1
ρ=8000.0-0.493T for T ≥ A1
ρ=7850-0.342T for T ≤ A1

We note here that equilibrium thermodynamics are strictly inappropriate for the present work, but the effects of this approximation on the results are likely small. We further note that the spike in heat capacity associated with the phase transformation at A1 was omitted. We also omitted heating or cooling effects associated with the latent heat of phase transformation during rapidly heated experiments near A1, although no significant temperature excursions were observed in the data.

Dynamic strain aging in this model is treated as an additional component to the yield and saturation stresses and hardening modulus that occur within a finite temperature range, the peak value of which depends on strain rate. The shape of the thermal softening data in the DSA range suggests the effect can be captured using a Gaussian peak function, which is written as follows:

**Table:** 

	${\hat{\tau }}_{y,DSA}={\hat{\tau }}_{y,0,DSA}\;exp\left[-\frac{{\left(T-T_{\mu }\left(\dot{\gamma }\right)\right)}^{2}}{\delta^{2}}\right]$ ${\hat{\tau }}_{s,DSA}={\hat{\tau }}_{s,0,DSA}\;exp\left[-\frac{{\left(T-T_{\mu }\left(\dot{\gamma }\right)\right)}^{2}}{\delta^{2}}\right]$ $\theta_{DSA}=\theta_{0,\;DSA}\;exp\left[-\frac{{\left(T-T_{\mu }(\dot{\gamma })\right)}^{2}}{\delta^{2}}\right]$	(13)

Four additional fitting parameters were introduced: ${\hat{\tau }}_{y,0,DSA}$, ${\hat{\tau }}_{s,0,DSA}$, $\theta_{0,\;DSA}$ and *δ*, which affect the amplitude of the DSA effect on yield, saturation, and hardening and the width of the temperature range where these effects are active. For temperatures below A1, these terms were added to the previous values of yield stress, saturation stress, and hardening modulus:

**Table:** 

	${\hat{\tau }}_{y}={\hat{\tau }}_{y}+{\hat{\tau }}_{y,DSA}$ ${\hat{\tau }}_{s}={\hat{\tau }}_{s}+{\hat{\tau }}_{s,DSA}$ $\theta =\theta +\theta_{DSA}$	(14)

**Fig. 3:**
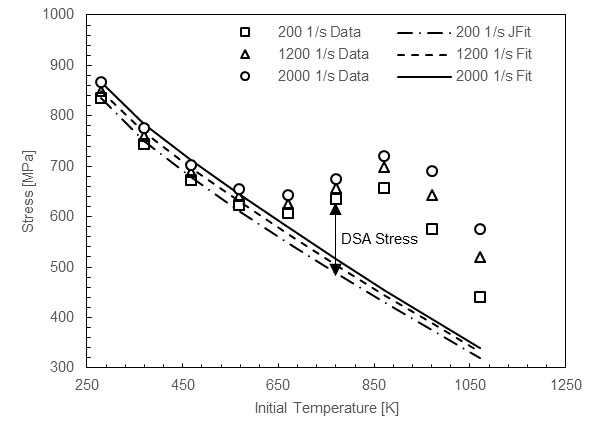
DSA stress magnitude estimates from literature data (strain = 0.4) [20].

The variation with strain rate of the temperature where DSA peak stress occurs ($T_{\mu }\left(\dot{\gamma }\right)$) was calibrated using available literature data. DSA stress magnitude was estimated from flow stress-temperature plots in a consistent way by fitting a the JC thermal softening model at low temperatures and extrapolating the fit into the DSA region at higher temperatures. Figure 3 shows an example of the method applied to literature data [20]. This method was also used to estimate the effect of strain rate on the peak magnitude of the DSA effect.

**Fig. 4:**
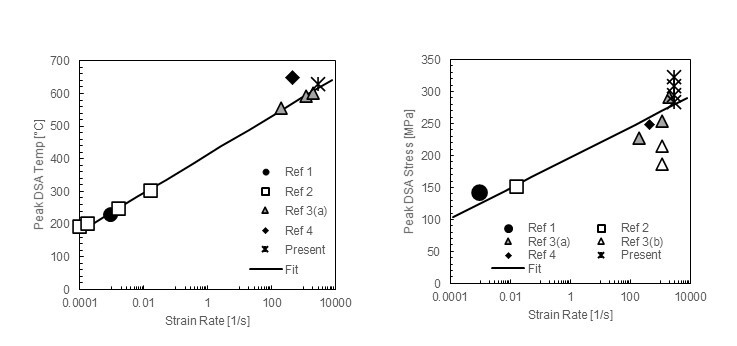
Effect of strain rate on peak temperature (left) and magnitude (right) of DSA stress effects on carbon steels. Ref. [16]: AISI 1004, ε = 0.113; Ref. [19]: AISI 1035, ε = 0.05; Ref. [20]: AISI 1018, (a) ε = 0.4, (b) ε = 0.1 to 0.2; Ref. [36]: AISI 1018, ε = 0.1; this study: AISI 1018, 1045, 1075, ε = 0.1 [4].

Figure 4 shows the variation in apparent peak temperature (left) and magnitude (right) of DSA effects from the present study and literature data. The data include different carbon contents and different levels of plastic strain, allowing some confidence in the generality of this correction but also pointing out the scatter in peak stress magnitudes seen in the data.

The change in peak DSA temperature with strain rate was captured by the following empirical fit shown in Fig. 4:

**Table:** 

	$T_{\mu }\left(\dot{\gamma }\right)=414+25.668\;ln\left(\frac{\dot{\gamma }}{\sqrt{3}}\right)$	(15)

The effect of strain rate on the magnitude of the DSA effect was handled similarly and was used to modify the value of all three hardening-related parameters (${\hat{\tau }}_{y,DSA}$, ${\hat{\tau }}_{s,DSA},\;$and $\theta_{DSA}$) to capture this behavior, using the fit of data presented in Fig. 4, as follows:

**Table:** 

	$F\left(\dot{\gamma }\right)=0.65+0.031\;ln\left(\frac{\dot{\gamma }}{\sqrt{3}}\right)$	(16)
	${\hat{\tau }}_{y,DSA}\left(\dot{\gamma }\right)=F\left(\dot{\gamma }\right){\;\hat{\tau }}_{y,0,DSA}\;exp\left[-\frac{{\left(T-T_{\mu }\left(\dot{\gamma }\right)\right)}^{2}}{\delta^{2}}\right]$ ${\hat{\tau }}_{s,DSA}\left(\dot{\gamma }\right)=F\left(\dot{\gamma }\right)\;{\hat{\tau }}_{s,0,DSA}\;exp\left[-\frac{{\left(T-T_{\mu }\left(\dot{\gamma }\right)\right)}^{2}}{\delta^{2}}\right]$ $\theta_{DSA}\left(\dot{\gamma }\right)=F\left(\dot{\gamma }\right)\;\theta_{0,\;DSA}\;exp\left[-\frac{{\left(T-T_{\mu }(\dot{\gamma })\right)}^{2}}{\delta^{2}}\right]$	(17)

While simplistic, this correction helps to avoid a large overprediction of DSA effects at low strain rates while maintaining a good fit of the behavior at high strain rates. We note that DSA effects measured with the NIST pulse-heated Kolsky bar may be artificially increased by possible friction effects because the electrical conduction through the bar/sample interface limits options for additional interface lubrication. As a result, the corrected parameters will underpredict the NIST data by a small amount, which may compensate for this issue to a degree.

The assembled model for the calculation of stress as a function of temperature, strain, and strain rate is:

**Table:** 

	${\hat{\tau }}_{T,\gamma ,\dot{\gamma }}={\hat{\tau }}_{y}+{\hat{\tau }}_{hardening}$ ${\hat{\tau }}_{hardening}=\;{\hat{\tau }}_{\gamma -\Delta \gamma }+\frac{\partial \hat{\tau }}{\partial \gamma }\Delta \gamma ={\hat{\tau }}_{\gamma -\Delta \gamma }+\theta \frac{{\hat{\tau }}_{s}-{\hat{\tau }}_{\gamma -\Delta \gamma }}{{\hat{\tau }}_{s}-{\hat{\tau }}_{y}}\Delta \gamma$	(18)

Normal (uniaxial) stress, strain, and strain rate data from compression experiments were converted to shear stress, shear strain, and shear strain rate using the Von Mises criterion:

**Table:** 

	$\tau =\frac{\sigma }{\sqrt{3}}$ ; $\gamma =\varepsilon \sqrt{3}$ ; $\dot{\gamma }=\dot{\varepsilon }\sqrt{3}$	(19)

In these relations, the left-hand sides are shear values and the right-hand sides are normal values.

### 2.1 Fitting Approach

The fitting procedure began with the low-temperature region (*T* < 400 °C) where DSA effects have yet to appear in the high-strain-rate data. We note that all fits of dynamic data were obtained using adiabatic temperatures, which were calculated using Eq. (12), such that the effects of strain hardening and thermal softening were fit simultaneously. Further, the experimental temperatures were adjusted for thermal gradients in the rapidly heated experiments [4] to further reduce the already small effects on the flow stresses. The initial fit was heavily weighted to the room-temperature experiments at high strain rate (>1000 1/s) because these data are the most relevant to machining since the workpiece material begins at low temperature before it flows through the primary shear zone. Low-strain-rate tests at room temperature and the heated tests at high strain rate were weighted less by assigning a fit weight of 0.25 for the low-strain-rate, room-temperature tests versus 1.0 for the high-strain-rate, room-temperature tests. The initial fit determined the values for nine parameters: *y_0_*, *y_∞_*, *s_0_*, *s_∞_*, *k*, *y_1_*, *y_2_*, *C_sat_*, and *θ*. Next, dynamic data between 400 °C and A1 were used to fit the four DSA parameters: ${\hat{\tau }}_{y,0,DSA}$, ${\hat{\tau }}_{s,0,DSA}$, $\theta_{0,\;DSA},$ and *δ*, with equal weighting of all experiments in this range. In this step, the mean DSA peak temperature (*T_μ_*) was also fit as a parameter, and this fit value was then used along with literature data to calibrate Eq. (15) and Eq. (16). Data above A1 were fit separately from data below A1; therefore, it was most convenient to implement the model in a finite element code via a lookup table. It was assumed that pearlite dissolved completely above A1, and the microstructure consisted of a nonequilibrium mixture of austenite having higher-than-equilibrium carbon content and excess ferrite compared to equilibrium. These data were used to find new values of *y_0_*, *y_∞_*, *s_0_*, *s_∞_*, *k*, and *θ* with equal weighting of all experiments, noting that DSA parameters were not needed after the phase transition. The rate sensitivity parameters were retained from the low-temperature fit because it is very difficult to measure the rate sensitivity of the metastable post-A1 material over wide ranges of strain rate, since slow mechanical tests provide time for further structural evolution. Finally, for tests above 10 1/s, adiabatic temperatures were evaluated using Eq. (12) prior to determining the fit constants via an iterative procedure.

Experimental conditions for the stress-strain curves used to generate the fit coefficients are summarized in Table 2. The material is annealed 1045 steel with a fine ferrite-pearlite microstructure that is described elsewhere [4]. Since the strength of carbon steel can vary significantly depending on the material microstructure after thermomechanical processing (size, shape, and distribution of precipitates and grains, and initial dislocation structure), the present set of model fit coefficients is only relevant for similarly processed 1045 steel. The maximum strains obtained in the experimental data are generally lower than those associated with machining processes, where strains can greatly exceed 1. This is a general limitation of uniaxial compression testing, where friction effects can become significant at strains larger than 0.5 [37]. Load-unload-reload methods or special end-milled specimens are used to achieve higher strains with reduced friction effects and may be useful for room-temperature testing, but neither method is practical for rapid electrically heated compression testing.

**Table 2:** Kolsky bar experiments used for model parameter fits. Bold indicates data used for low-temperature fit; italics indicate data used for DSA fit; gray fill indicates data used for post-A1 temperature fit.

Initial Temperature [°C]	Average Strain Rate [1/s]	Maximum Strain	Initial Temperature [°C]	Average Strain Rate [1/s]	Maximum Strain
**23**	**0.001**	**0.5**	*467*	*1 750*	*0.36*
**23**	**1**	**0.5**	*538*	*1 820*	*0.33*
**23**	**204**	**0.5**	*567*	*1 630*	*0.35*
**23**	**4 500**	**0.6**	*592*	*1 570*	*0.35*
**23**	**8 000**	**0.6**	*616*	*1 540*	*0.36*
**23**	**16 000**	**0.9**	*643*	*2 030*	*0.32*
**64**	**1 200**	**0.22**	*656*	*1 760*	*0.36*
**102**	**2 270**	**0.30**	711	2 720	0.42
**103**	**1 280**	**0.23**	746	3 020	0.43
**109**	**1 390**	**0.23**	766	3 060	0.41
**118**	**2 250**	**0.31**	793	3 470	0.43
**152**	**1 650**	**0.28**	832	3 640	0.44
**176**	**2 680**	**0.33**	894	3 730	0.43
**318**	**1 840**	**0.29**	919	3 990	0.48
**379**	**2 130**	**0.31**	954	3 790	0.43
*430*	*1 950*	*0.39*	1007	4 190	0.47

## 3 Fit Results and Discussion

**Fig. 5:**
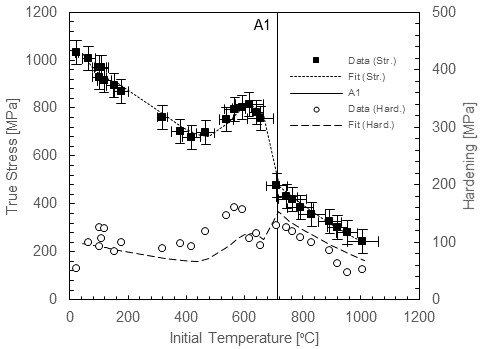

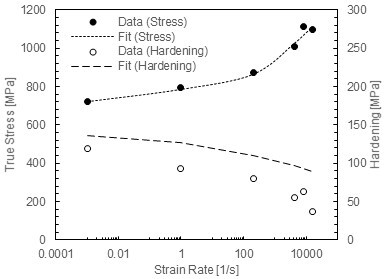
Replot of data from Fig. 1 showing model fit.

**Fig. 6:**
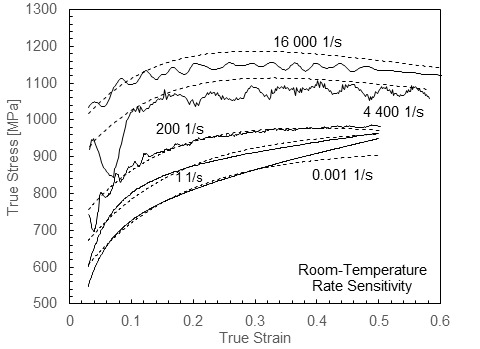

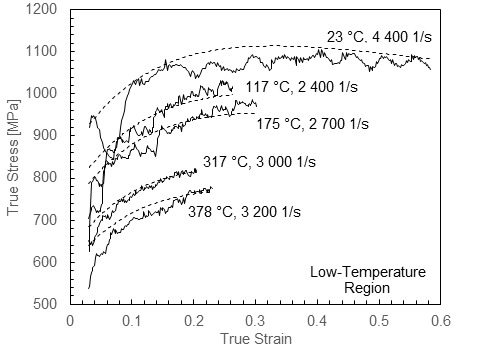

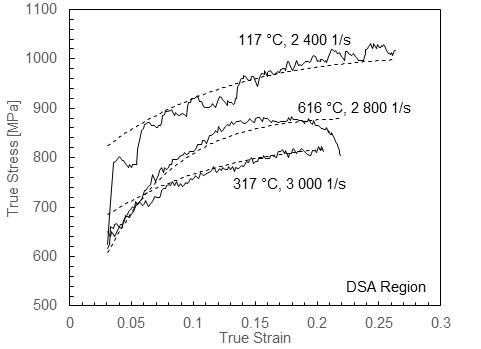

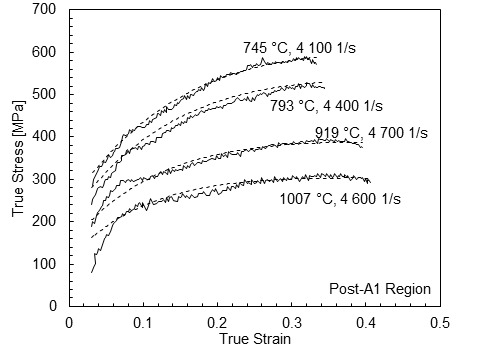
Selected experimental stress-strain curves and associated fits.

Fits were performed using the minimize function available in SciPy [38] with the Nelder-Mead solver [39]. Table 3 lists the fit coefficients for the low-temperature region, including strain rate sensitivity, the DSA region, and finally the post-A1 region for 1045 steel. These coefficients determined the normalized shear stress-strain behavior using Eqs. (7) to (11), Eq. (13), Eq. (14), and Eq. (17), and these values were converted to normal stress-strain values using Eq. (19). Figure 5 replots the data of Fig. 1 along with fits of flow stress at a strain of 0.1 and hardening between strains of 0.075 and 0.2. Figure 6 plots selected stress-strain curves and the associated fits to give a more detailed impression of the quality of the fits over the entire range of experimental strains measured. The model tracked the flow stress very well at 0.1 strain through the entire range. Hardening was generally underpredicted in the DSA region and overpredicted at room temperature. Experimental hardening values were determined by fitting each stress-strain curve with a simple power-law function and calculating the hardening values from the fits, in order to eliminate the influence of the oscillations in the experimental stress-strain curves. Often, this method underpredicted the experimental hardening, exaggerating the difference between the model hardening and data hardening. A better impression of the capacity of the model to capture the complex hardening behavior can be obtained from Fig. 6. Overall, the model demonstrated an ability to mimic the complex hardening trends exhibited by carbon steel over wide ranges of strain rate and temperature due to DSA and phase transformation effects. Finally, to implement the model in a machining simulation, where temperatures during adiabatic deformation can cross the A1 boundary between the two fits, a suitable bridging function is required to create a smooth, continuous transition between the fits. Further research is needed to develop the data and models necessary to capture the kinetics of the transition in a quantitative way.

**Table 3:** Fit coefficients for the-low temperature, DSA, and post-A1 regions of 1045 steel.

Region	*y_0_*		*y_∞_*	*s_0_*		*s_∞_*	*k*		*y_1_*		*y_2_*	*C_sat_*		*θ*
<400 °C	0.00406		0.0	0.00726		8.04e−7	0.748		0.0148		0.0555	2.11		0.0100
>712 °C	0.00574		0.0	0.0161		0.000183	1.32		0.0148		0.0555	1.01		0.0168
DSA Region		${\hat{\tau }}_{y,0,DSA}$			${\hat{\tau }}_{s,0,DSA}$			*θ_DSA_*		*T_μ_*			*δ*	
		3.15e−7			0.00342			0.0371		635 °C			113.27	

## 4 Comparison with Literature Machining Models for Carbon Steel

**Fig. 7:**
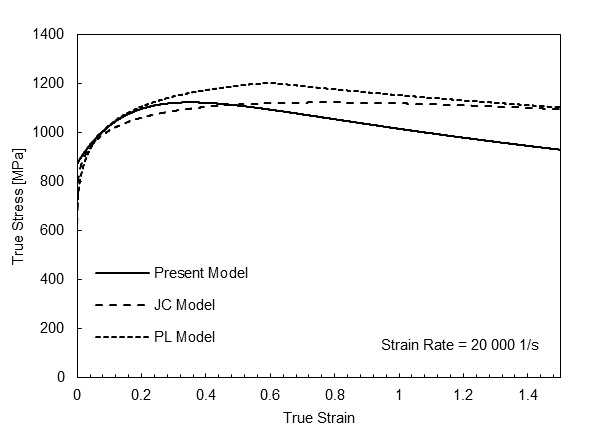
Adiabatic stress-strain curves at an initial temperature of 23 °C and a strain rate of 20 000 1/s.

We compared the current model with two previous models described in the literature that have been used to simulate 1045 carbon steel machining. The first is an unmodified JC model [34] (labelled “JC”) with coefficients determined from externally heated compression tests at various strain rates and temperatures up to 600 °C. The second model, labeled “PL,” is based on power laws for strain and strain rate hardening and thermal softening and contains additional parameters to mark stress saturation and DSA effects (referred to as blue brittleness in machining) [13]. The PL model was calibrated with literature data, primarily high-strain-rate heated compression data [20], and the model was developed for a carbon content slightly higher than the present work (0.55% C steel). Figure 7 plots adiabatic compression stress-strain curves generated by the JC and PL models and the present model under the machining conditions investigated in Ref. [34] (initial temperature of 23 °C, a strain rate of 20 000 1/s, and a maximum plastic strain of 1.5). We note that this condition is beyond the limits of the data used to calibrate the model parameters in all cases and is thus an extrapolation of every model. Comparing the current model to previous ones, we note that the current model indicates a smooth but noticeable peak in the adiabatic curve near a plastic strain of 0.5, followed by strain softening, while the PL model shows a sharper transition as it abruptly ends strain hardening via a critical strain parameter, with a value here of 0.6. The JC model shows a much more gradual transition to strain softening over the range of strains examined. The onset of strain softening is quite important in machining simulations because this point determines the onset of shear localization in predicted chip morphologies in two-dimensional orthogonal cutting models for some materials such as Ti-6Al-4V [40]. We note, however, that in machining annealed 1045 steel, the chips are usually continuous rather than serrated, unless very high cutting speeds are used [41].

**Fig. 8:**
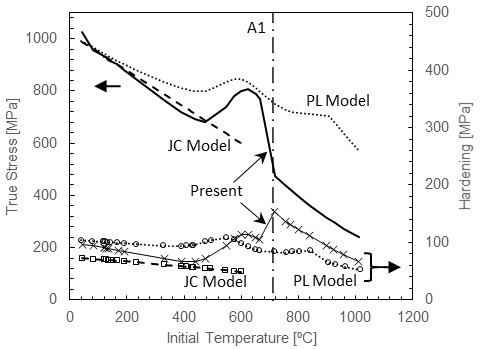

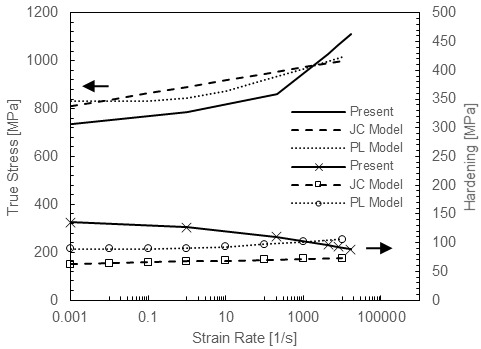
Comparison of present model fit with two fits for similar steels described in the machining literature. Stresses are indicated at a plastic strain of 0.1, and strain hardening values were evaluated between plastic strains of 0.075 and 0.2.

Figure 8 presents the temperature sensitivity of the flow stress at high strain rate (3500 1/s) and the rate sensitivity at room temperature predicted by the present model along with the JC model and the PL model. The evolution of hardening with temperature and strain rate is also presented in the plots, computed between plastic strains of 0.075 and 0.2. The complex hardening evolution with temperature of 1045 steel up through the DSA region was not perfectly captured by the current model, but the trends are correct, unlike results for the basic JC model, which had too few parameters to capture such behavior. The PL model was modified to capture DSA effects using a polynomial function, and, as such, it captured more of the complex hardening behavior exhibited by carbon steels. Interestingly, it has been noted that better predictions of cutting experiments were obtained without this DSA modification [13]. This model also employed a delayed transition in thermal softening due to austenite transformation, and at low temperatures, the thermal softening rate was less steep compared to the other models, motivated by better predictions of cutting experiments. However, while improved agreement is a powerful argument, numerous other factors can influence the outcome aside from the material model, including friction, thermal transport, and mesh resolution. Regarding room-temperature strain rate sensitivity, both the present model and the PL model captured the transition in rate sensitivity observed in experiments on steels, whereas the unmodified JC model did not, as expected. The evolution of hardening with strain rate remained constant with the JC model, trended upward in the PL model, and trended downward in the present model, the latter of which accurately represents the observed behavior.

## 5 Conclusions

The modified PTW model described in this work captures the complex flow stress and hardening behavior of 1045 ferrite-pearlite carbon steel over a wide range of strain rates and temperatures, as observed in rapidly heated Kolsky bar experiments. The Voce hardening model used here employs more fitting parameters (6) than either the Johnson-Cook model hardening (2) or power-law model hardening (2 or 3), giving greater flexibility to capture complex hardening brought about by dynamic strain aging and phase transformation, although separate fits are used to capture phase change effects. A notable consequence of the more complex hardening model used here is that it naturally captures the onset of flow softening due to adiabatic heating that is believed to govern the onset of serrated chip formation in orthogonal machining simulations. The new model also employs the two-stage strain-rate hardening model developed for low-carbon steel [10], requiring two fitting parameters rather than one. Finally, the new model employs a Gaussian function to capture dynamic strain aging effects, including the effect of strain rate on the peak effect temperature, at the cost of four additional fitting parameters. Future work will include a two-stage kinetics model to explicitly capture the effects of time and temperature on austenite growth into pearlite colonies and then ferrite, if this aspect is found to be relevant for simulating practical machining processes. A further modification is planned to capture the effect of carbon content on flow stress and hardening evolution for hypoeutectoid steels, which exhibit many similarities, and some notable differences, to the present 1045 steel.
